# Malaria in overseas labourers returning to China: an analysis of imported malaria in Jiangsu Province, 2001–2011

**DOI:** 10.1186/1475-2875-13-29

**Published:** 2014-01-25

**Authors:** Yaobao Liu, Michelle S Hsiang, Huayun Zhou, Weiming Wang, Yuanyuan Cao, Roly D Gosling, Jun Cao, Qi Gao

**Affiliations:** 1 Jiangsu Institute of Parasitic Diseases, Key Laboratory of Parasitic Disease Control and Prevention (Ministry of Health), Jiangsu Provincial Key Laboratory of Parasite Molecular Biology, Wuxi, Jiangsu, People’s Republic of China; 2 Medical College of Soochow University, Suzhou, People’s Republic of China; 3 Malaria Elimination Initiative, Global Health Group, University of California, San Francisco (UCSF), San Francisco, CA, USA; 4 Department of Pediatrics, UCSF Benioff Children’s Hospital, UCSF, San Francisco, CA, USA

**Keywords:** Imported malaria, Overseas labourer, Exported labourer, Investment, Jiangsu Province, China, Africa, Migrant

## Abstract

**Background:**

While great success in malaria control has been achieved in China, imported malaria has become a major challenge in the context of malaria elimination. This retrospective study describes the epidemiological profile of imported malaria and identifies the at-risk population during the period of 2001–2011 in Jiangsu Province.

**Methods:**

Data on imported malaria cases in Jiangsu Province from 2001 to 2011 were collected from the infectious disease surveillance system and case investigation reports. Epidemiological trends were described and correlations between trends in exported labour and malaria imported from other countries were explored.

**Results:**

From 2001 to 2011, 918 malaria cases and six malaria deaths were due to malaria imported from other countries, accounting for 12.4% of all malaria cases and 100% of all malaria deaths. During this time period the annual number of indigenous cases decreased from 1,163 to 13 while the number of imported cases increased from 86 to 366. The relative proportion of cases imported from other countries *versus* other provinces also increased from 0.0% (0/86) to 97.0% (350/361). The most affected demographic groups were males (897 cases, 97.7%) and adults (20–50 years old: 857 cases, 93.4%). All 918 cases had a recent travel history to malaria-endemic areas and the main purpose for travel was overseas labour (848 cases, 92.4%). The cases were mainly acquired from African countries (855 cases, 93.1%). *Plasmodium falciparum* was the most common species (733 cases, 79.8%). The increase in malaria cases imported from other countries was associated with the growth of investment to Africa from Jiangsu (R^2^ = 0.8057) and the increasing number of exported labourers to Africa from Jiangsu (R^2^ = 0.8863).

**Conclusions:**

From 2001 to 2011 in Jiangsu Province, there was a consistent increase in the number of malaria cases imported from other countries while the number of locally acquired cases sharply declined. This trend may be ascribed to the increasing investment from China to Africa and the rising number of Chinese labourers working in Africa. Preventative efforts should be targeted to this high-risk group and the surveillance and response system should be strengthened to prevent local resurgence in Jiangsu.

## Background

Malaria remains one of the major public health problems in the world. There are more than 100 malaria-endemic countries and almost one million deaths annually. The main endemic areas are sub-Saharan Africa, South America, the Pacific islands and Southeast Asian countries [1]. Due to global economic integration, large numbers of people travel to malaria-endemic countries for trade, tourism, labour and other purposes. Subsequently, importation of malaria from highly malaria-endemic areas into lower or non-endemic countries is inevitable [2,3]. Approximately 125 million international travellers visit malaria-endemic countries yearly and over 10,000 cases are reported after returning home, according to the World Health Organization [4]. This situation makes malaria not only a threat to non-immune travellers, but also among local populations where those travellers live. For countries or areas that are near to or have already achieved elimination, imported malaria is a risk for resurgence or re-introduction of malaria.

Jiangsu Province is located in eastern China and had an estimated total population of 79 million people in 2011 [5]. The main malaria vectors are *Anopheles sinensis* with *Anopheles anthropophagus*[6]. Historically, malaria transmission was unstable and prone to large outbreaks. There were two peak epidemic periods in the 1960s and 1970s during which time about 10 million malaria cases were reported each year and the annual incidence was as high as 250/1,000 population. *Plasmodium vivax*, *Plasmodium falciparum* and *Plasmodium malarie* were endemic in Jiangsu, with the dominant species being *P. vivax*. Indigenous falciparum malaria was successfully eliminated in 1988 [7]. Indigenous vivax malaria in Jiangsu has been effectively controlled through comprehensive implementation of effective interventions and only 13 indigenous vivax cases were reported in 2011 [8]. In the context of the national malaria elimination programme, which was launched in 2010 and aims to achieve malaria elimination nationwide in China by 2020, Jiangsu province aims to interrupt local malaria transmission by 2015 [9,10].

In recent years, there has been a rise in imported malaria in Jiangsu Province, providing a threat to elimination goals. In order to address this new challenge, a better understanding of imported malaria is needed. In this study, the epidemiological profile and trends of imported malaria in Jiangsu province from 2001–2011 are described.

## Methods

### Study design and data collection

Utilizing routine surveillance data from the China Centres for Disease Control and Prevention (CDC), a retrospective analysis of imported malaria in Jiangsu Province from 2001 to 2011 was carried out. All malaria cases were diagnosed and treated according to the national policy. Laboratory confirmed cases were diagnosed by microscopy. All positive slides and 10% of negative slides are reviewed for confirmation by an expert microscopist at the provincial laboratory. Non-laboratory confirmed cases, including species classification of these cases, were diagnosed according to symptoms, response to therapy, any previous diagnosis of malaria, and predominate species in the geographic origin of infection.

Monthly case reports were collected through the China Information System for Disease Control and Prevention (CISDCP), the national internet-based disease reporting system. More detailed demographic and clinical data, as well as travel history, were obtained from follow-up case investigations. Based on Technical Scheme of China Malaria Elimination [11], cases were classified by local staff as indigenous (acquired within the province), imported from other provinces (travel within the previous month to another district in China with local cases reported), or imported from other countries (travel within the previous month to a malaria-endemic country). Otherwise, the case was classified as local. When the source was unclear, the infection origin was determined by a provincial or national expert team.

Annual data on investment to Africa, and the number of labourers exported from Jiangsu to Africa were collected from the Jiangsu statistic yearbook [5] and confirmed by the Department of Commerce of Jiangsu Province.

### Data management and analysis

Data were double entered into Microsoft Excel 2007 and then imported into SPSS17.0 to conduct the analysis. Characteristics of imported malaria were described according to geographical and temporal distribution, age and gender composition, travel purpose, country from where the infection was acquired, and *Plasmodium* species.

The trend lines for the malaria situation were generated by Moving Average (MA) method of Microsoft Excel 2007. The associations between the annual number of cases imported from other countries and annual number of exported labourers to Africa as well as annual investment to Africa from Jiangsu Province were analysed using linear regression analysis.

### Ethical considerations

The study was approved by the Institutional Review Board of Jiangsu Institute of Parasitic Diseases (IRB00004221), Wuxi, China.

## Results

### Epidemiologic profile of malaria in Jiangsu Province from 2001 to 2011

Over the 11 years from 2001 to 2011, a total of 7,421 malaria cases were reported in Jiangsu Province. Most of the cases were classified as indigenous (65.7%, or 4,877 cases); 21.9% (1,626 cases) were imported from other provinces of China and 12.4% (918 cases) were imported from other countries.

**Figure 1:**
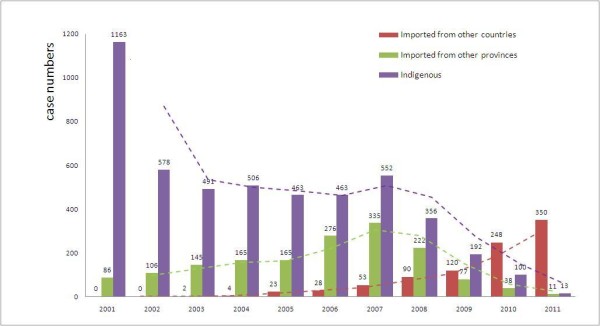
**Malaria situation in Jiangsu Province, 2001–2011.** The dashed lines and columns of different colours show the change trend of cases imported from other countries (red), cases imported from other provinces (green) and indigenous cases (blue), respectively.

The annual number of indigenous cases sharply declined from 2000 to 2001, after which it was relatively stable, and then declined steadily after 2007. Imported cases from other provinces increased from 2001 to 2007 then declined steadily afterwards. The annual number of cases imported from other countries increased dramatically over the study period, exceeding the number of cases imported from other provinces in 2009, and exceeding the number of indigenous cases in 2010 (Figure 1). The relative proportion of cases imported from other countries *versus* other provinces increased from 0.0% (0/86) in 2001 to 97.0% (350/361) in 2011.

### Demographic characteristics of malaria cases imported from other countries

**Figure 2:**
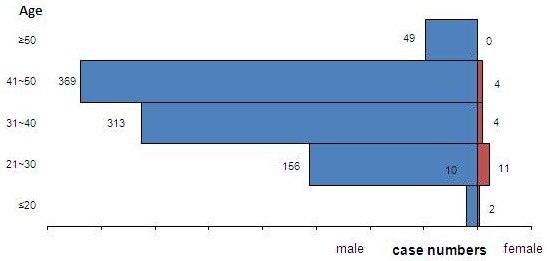
**Imported cases from other countries, by age and sex.**

**Figure 3:**
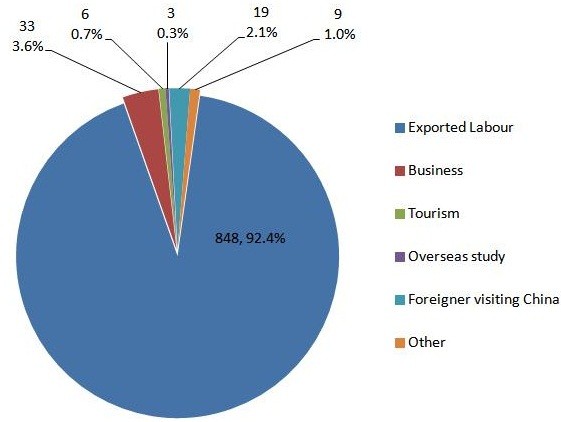
Travel purposes of malaria cases imported from other countries.

Of the 918 malaria cases imported from other countries, 857 cases (93.4%) were 20 to 50 years of age and the median age was 40 years (range: 11 to 69). Most cases occurred in males (897 cases, 97.7%) (Figure 2). Chinese citizens accounted for 898 cases (97.8%) and 20 cases (2.2%) were in foreigners. The main purpose for travel was labour (848 cases, 92.4%) (Figure 3).

### Clinical and laboratory characteristics of malaria cases imported from other countries

*Plasmodium falciparum* was the dominant species accounting for 754 cases (82.1%). *Plasmodium vivax* accounted for 128 cases (13.9%), *P. ovale* 14 cases (1.5%), *P. malariae* seven cases (0.7%), and 15 (1.6%) were not classified by species. Laboratory confirmation occurred in 815 cases (88.8%) and 103 cases (11.2%) were diagnosed clinically. *Plasmodium falciparum* diagnoses were more likely to be laboratory confirmed (687/754, 91%) than *P. vivax* cases (101/128, 78.9%). There were six deaths (0.7%) all due to *P. falciparum*.

Demographic and clinical details from malaria deaths are shown in Table 1. All malaria deaths occurred in men aged 29 to 49 years returning from overseas labour in Africa. Median time intervals were as follows: from arrival in China to symptom onset was five days (range: -8–11), from symptom onset to first health facility visit was one day (range: 0–8), from first health facility visit to malaria diagnosis was four days (range: 2–8), and from symptom onset to malaria diagnosis was 4.5 days (range: 2–12).

**Table 1:** Clinical and demographic details of malaria deaths

						**Time intervals (days)**			
**Case no**	**Year**	**Prefecture**	**Age (years)**	**Gender**	**Origin country**	**Arrival in China to symptom onset**	**Symptom onset to first health facility visit**	**First health facility visit to malaria diagnosis**	**Symptom onset to malaria diagnosis**
1	2004	Yangzhou	36	Male	Congo (Brazzaville)	3	3	8	11
2	2009	Suqian	49	Male	Angola	7	0	4	4
3	2010	Huaian	29	Male	Angola	11	2	2	4
4	2011	Lianyungang	32	Male	Ghana	-8^a^	8	4	12
5	2011	Nanjing	37	Male	Mozambique	2	0	5	5
6	2011	Taizhou	49	Male	Equatorial Guinea	11	0	2	2

^a^Symptom onset before arrival in China.

### Temporal and geographic characteristics of malaria cases imported from other countries

**Figure 4:**
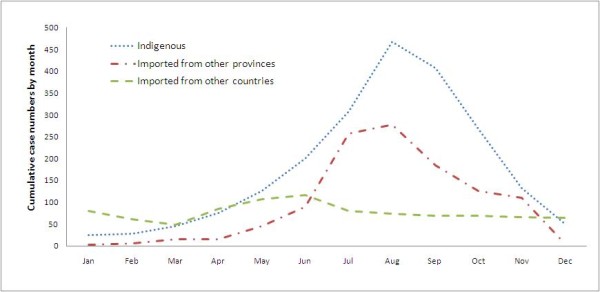
**Monthly distributions of malaria cases in Jiangsu Province, 2001–2011.**

Monthly reports of malaria cases imported from other countries showed no temporal pattern. In comparison, both indigenous cases and cases imported from other provinces of China peaked during the summer seasons (Figure 4).

**Figure 5:**
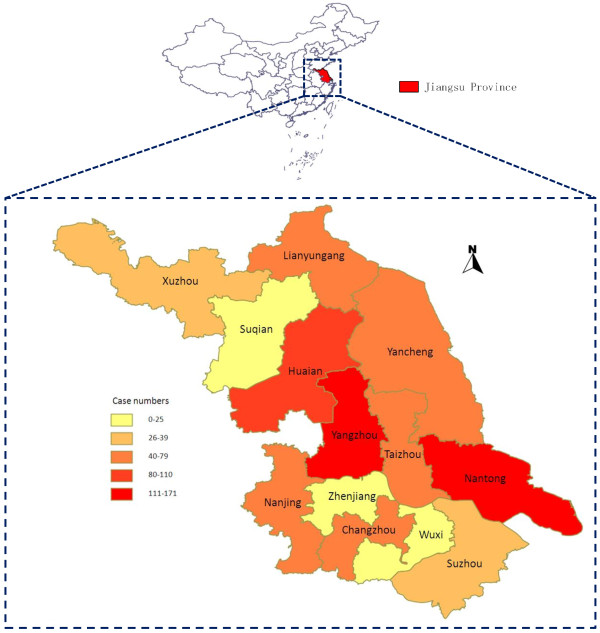
**Distribution of imported malaria cases from other countries, 2001–2011.** Case numbers are marked on the map by 13 prefectures of Jiangsu Province.

All 13 prefectures of Jiangsu Province reported malaria cases imported from other countries though cases were concentrated in Yangzhou (171 cases, 18.6%), Nantong (150 cases, 16.3%) and Huai’an (110 cases, 12.0%) (Figure 5). Most cases were acquired from Africa (855/918, 93.1%), with the top three countries from where malaria was imported being Angola (245 cases), Nigeria (219 cases) and Equatorial Guinea (146 cases). By species, most cases were also from Africa: *P. falciparum* cases (733/754, 97.2%), *P. vivax* (88/128, 68.8%), *P. ovale* (14/14, 100%) and *P. malariae* (7/7, 100%). The region with the second largest number of imported cases was Southeast Asia (32/918, 3.5%). These cases accounted for 24.2% (31/128) of all *P. vivax* cases (Table 2).

**Table 2:** **Malaria imported from other countries**, **by species and country**

**Origin country**	**Species**					**Total n = 918**
	***P. falciparum *** **n = 754**	***P. vivax *** **n = 128**	***P. ovale *** **n = 14**	***P. malariae *** **n = 7**	**Unclassified n = 15**	
**Africa**	**733 (97.2%)**	**88 (68.8%)**	**14 (100%)**	**7 (100%)**	**13 (86.7%)**	**855 (93.1%)**
Angola	219	17	1	3	5	245
Nigeria	185	27	6	0	1	219
Equatorial Guinea	130	10	5	0	1	146
Congo (Brazzaville)	21	1	0	2	0	24
Ghana	19	3	0	0	0	22
Gabon	19	1	0	1	0	21
Madagascar	15	0	0	0	2	17
Congo (Kinshasa)	12	4	0	0	1	17
Mozambique	14	1	1	0	0	16
Sudan	13	3	0	0	0	16
Niger	10	3	0	0	0	13
Guinea	11	0	0	0	0	11
Liberia	4	5	0	0	0	9
Uganda	8	0	0	0	0	8
South Africa	4	2	0	0	1	7
Togo	5	0	0	0	0	5
Malawi	3	1	0	1	0	5
Mali	4	0	0	0	0	4
Sierra Leone	4	0	0	0	0	4
Senegal	4	0	0	0	0	4
Cameroon	4	0	0	0	0	4
Côte d’Ivoire	2	1	1	0	0	4
Tanzania	3	0	0	0	0	3
Burkina Faso	2	0	0	0	0	2
Chad	2	0	0	0	0	2
Namibia	1	0	0	0	0	1
Rwanda	1	0	0	0	0	1
Kenya	1	0	0	0	0	1
Zambia	1	0	0	0	0	1
Ethiopia	0	1	0	0	0	1
Unclear Africa	12	8	0	0	2	22
**South Asia**	**0 (0.0%)**	**31 (24.2%)**	**0 (0.0%)**	**0 (0.0%)**	**1 (6.7%)**	**32 (3.5%)**
India	0	16	0	0	1	17
Pakistan	0	15	0	0	0	15
**Southeast Asia**	**6 (0.8%)**	**6 (4.7%)**	**0 (0.0%)**	**0 (0.0%)**	**1 (6.7%)**	**13 (1.4%)**
Myanmar	4	3	0	0	0	7
Indonesia	0	2	0	0	0	2
Laos	2	0	0	0	0	2
Cambodia	0	1	0	0	0	1
Thailand	0	0	0	0	1	1
**Oceana**	**4 (0.5%)**	**1 (0.8%)**	**0 (0.0%)**	**0 (0.0%)**	**0 (0.0%)**	**5 (0.5%)**
Papua New Guinea	4	1	0	0	0	5
**Latin America**	**0 (0.0%)**	**1 (0.8%)**	**0 (0.0%)**	**0 (0.0%)**	**0 (0.0%)**	**1 (0.1%)**
Bolivia	0	1	0	0	0	1
**Unknown**	**11 (1.5%)**	**1 (0.8%)**	**0 (0.0%)**	**0 (0.0%)**	**0 (0.0%)**	**12 (1.3%)**

### Increasing malaria cases imported from other countries associated with investment and labourers exported to Africa from Jiangsu Province

**Figure 6:**
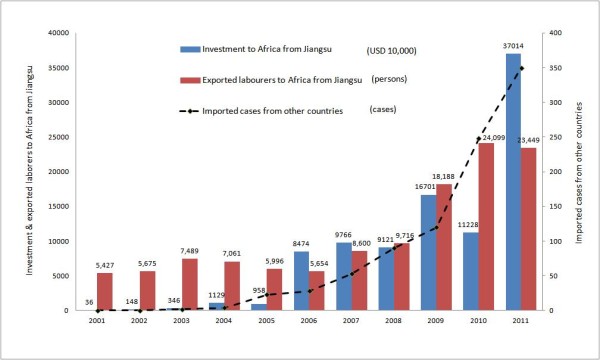
**The investment and labourers exported to Africa from Jiangsu Province, 2001–2011.** Annual number of imported malaria cases from other countries associated with the investment and labourers exported to Africa from Jiangsu Province.

Investment to African countries from Jiangsu Province increased over the study period and was related to the annual number of cases imported from other countries, R^2^ = 0.8057. The annual number of labourers exported from Jiangsu to Africa also increased over the study period and this trend corresponded with the increase in cases imported from other countries, R^2^ = 0.8863 (Figure 6).

## Discussion

With globalization and increased international movement, imported malaria has become an important public health issue. For countries that are approaching or have achieved elimination, imported malaria is a risk for resurgence or re-introduction [12]. This study reviewed the imported malaria situation in Jiangsu, a province of China where the malaria burden has decreased considerably and elimination is now the goal. Focusing on the 11-year period from 2001 to 2011, the annual number of indigenous cases in Jiangsu declined dramatically. The annual number of cases imported from other provinces showed a similar trend to the indigenous cases reflecting the decrease in malaria in other parts of China [13]. However, there was a consistent increase in the number of malaria cases imported from other countries into Jiangsu Province.

Most imported cases were due to *P. falciparum*, the species most commonly associated with severe disease and death. Prompt diagnosis and appropriate treatment are critical [14]. However, the diagnosis is frequently missed or delayed. Of 309 falciparum cases in 2011, six cases were diagnosed more than 30 days after the first symptoms of the malaria clinical attack and three patients died because of delayed anti-malarial treatment [8]. The rarity of *P. falciparum* and non-specific presentation pose challenges to health care workers in non-endemic settings [15]. Indeed, in Jiangsu Province, most physicians and healthcare providers, especially those at the primary level, lack the awareness and skills to manage falciparum malaria [16].

The potential for local transmission from imported malaria is a challenge for malaria elimination [17-19]. Outbreaks from imported cases have been reported in many settings where transmission was previously interrupted [20-22]. In some situations, such as recently occurred in Greece, there has been re-establishment of local transmission [23]. Although *P. falciparum* was eliminated from Jiangsu Province more than 20 years [7] and *P. vivax* has almost been eliminated (there were no locally acquired cases of malaria reported in 2012 [24]), historically the province was a highly endemic area. The malaria vector is still widely distributed throughout the province and environmental conditions may be suitable for transmission [25]. The low immunity in the population is a further risk for outbreaks, as is the relaxation of surveillance, which often happens as burden declines. In 2000, after a long period of stable, low endemic malaria, there was a large outbreak of vivax malaria in Sihong County related to a resurgence of malaria in nearby Anhui Province [26]. Although cases imported from other countries have not led to secondary cases, careful surveillance and further research to prevent the re-introduction of malaria by imported cases from other countries is required.

Most of the malaria cases imported from other countries came from Africa. This is largely due to the increased travel to Africa by Chinese nationals. Since the 2000 Forum on China-Africa Cooperation (FOCAC), there has been increased investment and foreign aid from China to Africa. In this context, investment from Jiangsu Province to Africa has greatly increased in recent years. In particular, exported labour for construction has increased as more companies are supporting infrastructure development [27]. The study showed that increased investment and increased number of exported labourers to Africa from Jiangsu is closely related to the increased number of cases imported from other countries during 2001 to 2011. Although the data on the number of overseas labourers returning to Jiangsu from Africa each year was not able to be obtained, it is expected that the trends would be similar to that of Chinese labourers going to Africa.

The risk of acquiring malaria varies amongst travellers [28]. In Jiangsu Province, malaria imported from other countries mostly occurs in adult males returning from overseas labour. In other malaria elimination settings, adult males have also been found to be the new high-risk group, compared to higher endemic settings where young children and pregnant women are the highest risk group for mortality and morbidity [29]. Labourers are susceptible for several reasons. First, they usually work outdoors on construction sites and live in poor housing conditions, putting them at high risk for mosquito bites. Second, labourers generally lack immunity to malaria, especially to *P. falciparum*. Third, exported labourers are generally poorly educated and lack awareness of risks of malaria and personal protection [30].

The majority of labourers are exported through labourer service or construction companies. These companies should be targeted for the development of prevention programmes, such as in improved housing, health education and prophylaxis. Programmes could also be targeted geographically. 46.9% of cases imported from other countries occurred in three prefectures: Yangzhou, Nantong and Huai’an (Figure 4). Nantong has a large construction industry [31]. In Yangzhou and Huai’an there are numerous intermediary companies offering labourer services. The CDC considered programmes that could be targeted temporally (e g, border screening during the holidays when Chinese nationals are returning home), but there was no seasonal pattern as has been reported in other settings [32].

Intersectorial cooperation between departments of health, education, security, commerce, travel, inspection, and quarantine can play an important role in the management of imported malaria. For example, the entry-exit inspection and quarantine agencies can carry out malaria screening for febrile travellers and then inform the local CDC of positive malaria cases. Other departments can cooperate with local CDC to carry out health education for labourers and others travelling to malaria-endemic countries.

## Conclusion

Imported malaria showed an increasing trend from 2001 to 2011 and it has become a major challenge for malaria elimination in Jiangsu Province. The main reason for the increase is the large number of exported labourers to malaria-endemic areas of Africa. Preventative measures should be targeted to high-risk groups and strengthened surveillance and response is needed to prevent local introduction of malaria.

## Competing interests

The authors declare that they have no competing interests.

## Authors’ contributions

YBL and JC conceived the study, YBL collected and analyzed the data and drafted the manuscript. MSH and RG contributed to data analysis and manuscript writing. HYZ contributed to the data interpretation and coordination. WMW contributed to data collection and interpretation. YYC contributed to data analysis and map drawing. JC and QG provided guidance and coordination throughout the entire process. All authors read and approved the final manuscript.
